# “WE DON’T AGE ALONE; WE AGE AS A COMMUNITY”: BARRIERS AND ENABLERS OF AGING WELL IN BLACK/AFRICAN AMERICAN OLDER ADULTS

**DOI:** 10.1093/geroni/igad104.3159

**Published:** 2023-12-21

**Authors:** Mushira Khan, John Holton, Ajla Basic

**Affiliations:** Mather, Evanston, Illinois, United States; University of Illinois Chicago, Chicago, Illinois, United States; Mather, Evanston, Illinois, United States

## Abstract

Eurocentric understandings of successful aging privilege optimal physical and cognitive health, absence of disease and disability, and active social engagement in later life. This marginalizes the experiences of diverse older adults who may live with chronic conditions but maintain a good quality of life, supported by factors such as spirituality, resilience, and close familial ties. Further, this approach is myopic because it fails to capture social determinants of health (SDOH) that influence the aging process over time. The Perceptions of Aging Well in Diverse Older Adults study is a 5-year series that seeks to identify: 1) micro-, meso- and macro-level factors that shape aging well in racial/ethnic minority older adults, and 2) strategies to support aging well in racial/ethnic minority older adults. This presentation highlights preliminary findings from a sub-study with Black/African American participants. In-depth, semi-structured interviews were conducted with 23 community-dwelling Black/African American adults age 50+ (14 females, 9 males; mean age = 71.2 years). Thematic analysis of the interview data showed that neighborhood safety, food insecurity, pharmacy deserts, adverse childhood experiences, structural racism, and everyday micro-aggressions shaped perceptions of aging well in powerful ways. Strong kinship bonds and a desire to leave a legacy of resilience and empowerment for younger generations motivated participants to engage in prosocial activities such as volunteering in local church communities. These findings suggest that along with addressing key SDOH, fostering generativity through teaching and mentoring, creative arts, and taking on community leadership roles may enhance subjective well-being in Black/African American older adults.

